# Variation in aeolian environments recorded by the particle size distribution of lacustrine sediments in Ebinur Lake, northwest China

**DOI:** 10.1186/s40064-016-2146-0

**Published:** 2016-04-19

**Authors:** Long Ma, Jinglu Wu, Jilili Abuduwaili

**Affiliations:** State Key Laboratory of Desert and Oasis Ecology, Xinjiang Institute of Ecology and Geography, Chinese Academy of Sciences, Urumqi, 830011 China; State Key Laboratory of Lake Science and Environment, Nanjing Institute of Geography and Limnology, Chinese Academy of Sciences, Nanjing, 210008 China

**Keywords:** Aeolian transportation, China, Ebinur Lake, Lake sediment, Particle size

## Abstract

Particle size analysis of lacustrine core sediments and atmospheric natural dust were conducted in the drainage area of Ebinur Lake in arid northwest China. Using a combination of ^137^Cs and ^210^Pb dating, a continuous record of aeolian transportation to the lake sediments and related factors over about the past 150 years was analyzed. Factor analysis revealed the particle-size distributions of riverine and aeolian sediments composed of the terrigenous materials of the lake deposits. Compared with the grain-size distributions of natural dust samples, the results showed that the coarser particle size fraction of lake sediments was mainly derived from the sediments that had experienced aeolian transport to the drainage surface, and the finer sediments came from hydraulic inputs. Then, the method of variations in particle-size standard deviation was used to extract the grain size intervals with the highest variability along a sedimentary sequence. The coarser grain-size populations dominated the variation patterns of the sedimentary sequence. During the last 150 years, strong intensity aeolian transportation occurred during three periods, 1915–1935, 1965–1975 and since the beginning of the 2000s. The climate was dry around 1910s–1930s in this region associated with the appropriate dynamic condition, which provided the enhanced source materials and wind power for the aeolian dust transport. Since 1950s, the climate controlled the foundation of aeolian dust transport, and the aeolian dust transport won’t be increased under the humid climate.

## Background

Long-term temporal perspectives provide an important part of understanding contemporary environmental change and related processes (Anderson 1995). Analyzing the environmental evolution, mutation events and factors that influenced them in the historical period will also provide a reference for future regional eco-environmental protection. Dust is recognized as an important physical and chemical flux within ecosystems (Lawrence and Neff 2009; McTainsh and Strong 2007), and dust storms are prevalent in arid regions (Xu et al. 2006). Currently, both the characteristics of modern dust storms (Wake et al. 1994; Wang et al. 2004) and the mechanisms that control them (Wang et al. 2006) have been extensively studied. However, the changes of dust storms in the historical period and the factors influencing that change also need to be analyzed under the context of human activity and natural factors.

Lacustrine sediments have the composition of autochthonous and allochthonous materials that provide information about past environmental change in the surrounding watersheds (Last and Smol 2006; Overpeck et al. 1997). Particle-size of terrigenous materials in lake sediments have been widely used as environmental indicators in sedimentary researches (Peng et al. 2005; Qiang et al. 2007; Zhong et al. 2010). In arid regions, variations in sediment transport, such as hydraulic and aeolian transport, cause the distribution of grain-size to be polymodal and represent different transport and depositional processes (Sun et al. 2002). Some researchers used Weibull (Sun et al. 2002) and unimodal lognormal distributions (Qin et al. 2005; Xiao et al. 2009), respectively, to decompose multimodal grain-size distributions. End-member mixing analysis also used to extract environmentally sensitive populations of particle size data (Weltje 1997). Boulay et al. (2003) used the variations of standard deviation to obtain the grain size intervals with the highest variability along a sedimentary sequence. These methods provided powerful tools for decomposition of particle-size distributions that inferred the different transport and depositional processes.

In this study, we focused on the distribution of particle size in Ebinur Lake sediments. Our objectives were: (1) to identify the history of aeolian transport over the past ~150 years, and (2) to conduct a regional comparison and reveal the possible factors influencing the variation in aeolian transport.

## Methods

### Sample collection and analysis

**Fig. 1:**
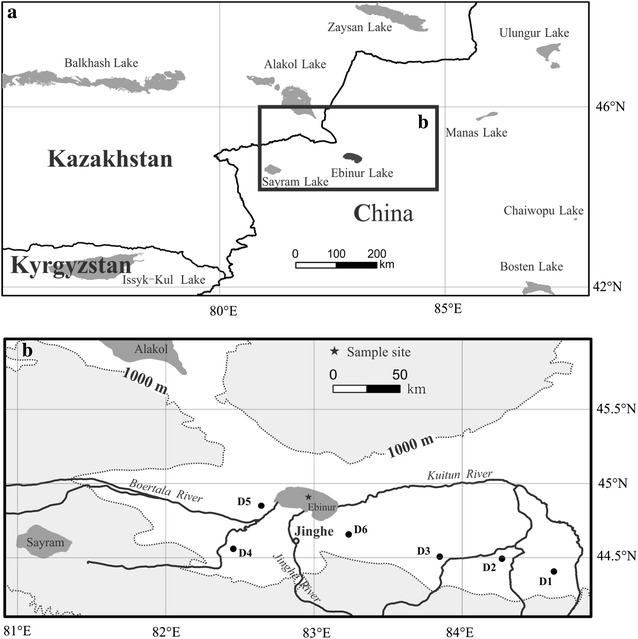
Vicinity map of the regions of Ebinur Lake (**a**), and the sites of core sediment and dust samples (**b**)

Ebinur Lake, closed lake in northwestern China, lies near the border to Kazakhstan and at the southeast end of the Dzungaria Gate. The catchment of Ebinur Lake drains an area of 50,321 km^2^. The lake water depth averages 1.2 m with a maximum 3.5 m (Wu et al. 2009). A 50-cm short sedimentary core (AB01) was obtained from north-central part of Ebinur Lake using a piston-percussion corer with a 60-mm inner diameter perspex tubes in 2011 (Fig. 1). In this study, all sediments samples were sectioned into 1 cm intervals. The subsamples were kept in plastic bags and stored at 4 °C before being analyzed.

Passive dustfall collectors were used to trap aeolian dust samples in the Ebinur drainage area (Liu et al. 2011). Traps consisted of polyethylene cylinders with a diameter of 15 cm and depth of 30 cm mounted vertically on posts about 3 m above the ground and no specific trapping mechanisms. Dustfalls was brushed off from the collectors into prepared clean small bags. Subsamples for grain-size distribution measurements were extracted by a quartering method that reduces a sample by successively mixing, dividing into quarters and keeping two opposite quarters of the sample.

### Laboratory analysis

^210^Pb and ^137^Cs were detected by direct gamma spectrometry using an Ortec HPGe GWL series, well-type, coaxial, low background and intrinsic germanium detector (Wu et al. 2009). Grain-size distribution measurements of all samples from lake sediments and aeolian dusts were carried out on a Malvern Mastersizer 2000 equipped with a Hydro 2000 MU dispersion unit with a measurement range of 0.02–2000 μm. Prior to particle size analysis, with 10–20 ml of 30 % H_2_O_2_, samples were pretreated to remove organic matter, and to remove carbonates with 10 ml of 10 % HCl. Deionized water (2 l) was added and 24 h standing to remove the acid. Finally, the sample residue was treated with 10 ml of 0.05 M (NaPO_3_)_6_, and then, placed on an ultrasonic vibrator for 10 min sonication to facilitate dispersion before particle size analysis. A Malvern Mastersizer-2000 analyzer equipped with a Hydro 2000 MU dispersion unit, from Malvern Instruments Ltd. (Malvern, Worcestershire, UK) was used to conduct the particle size. The pump speed was set at 2000 rpm, and the refractive index and absorption parameter were 1.520 and 0.1, respectively. The sample was added till obscuration range was within 10–20 %. The Mastersizer 2000 automatically yields the percentages of the related size fractions of a sample with a relative error scale of less than 1 %.

## Results

**Fig. 2:**
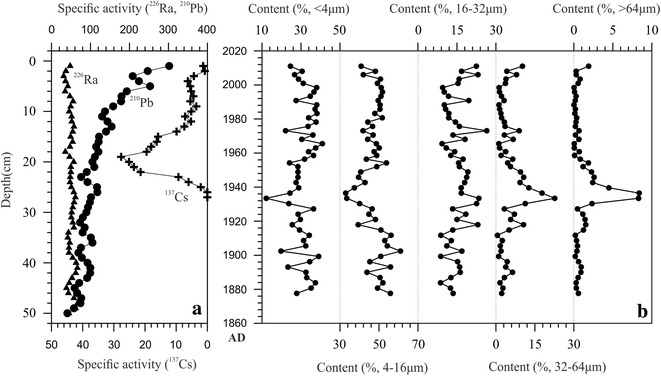
The specific activity of ^137^Cs, total ^210^Pb and ^226^Ra versus depth in sediment cores of Ebinur Lake (**a**) and the related particle size distribution of the sediment cores (**b**)

Figure 2a shows the distribution of ^210^Pb and ^137^Cs activities. The unsupported ^210^Pb activity (^210^Pb_ex_, ^210^Pb_ex_ = total ^210^Pb − ^226^Ra). The ^210^Pb_ex_ decreased from 250 Bq/kg at surface to zero at nearly 49 cm (Fig. 2a). A constant rate of supply model was used to calculate the date for each core sample (Appleby 2001). The beginning of ^137^Cs activity was appointed 1954 and the peak value was the 1963 fallout maximum from atmospheric testing of nuclear weapons (Pennington et al. 1973), which was consistent with the ^210^Pb chronology. Figure 2b provides a plot of the sedimentary data versus geologic age.

**Fig. 3:**
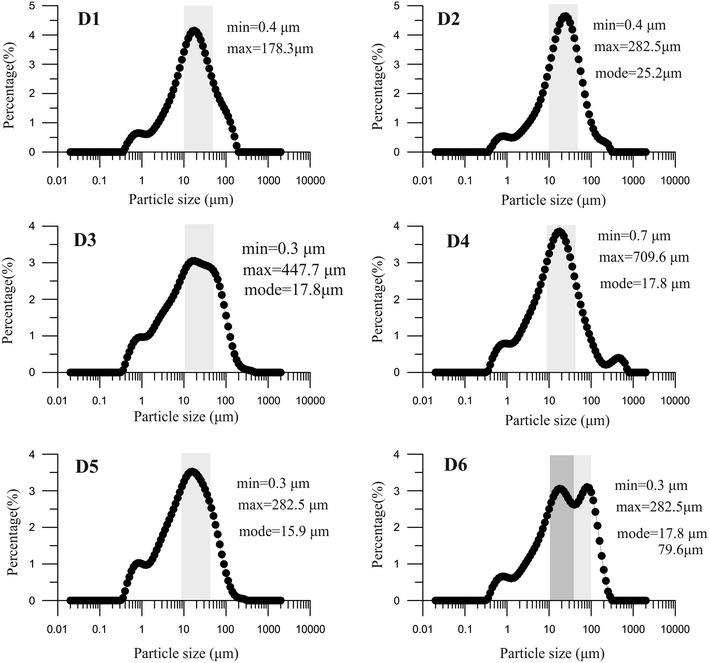
Particle size distribution of the atmospheric dust samples (**D1**–**D6**) from Ebinur region

Sediment particle size fractions of (<4 μm), (4–16 μm), (16–32 μm), (32–64 μm) and (>64 μm) were generally about 31.1, 47.5, 15.2, 5.1, 1.1 %, respectively. Figure 2b presents values of particle size plotted versus depth, which show strong variations in particle size occur at a depth of about 30 cm. Long-term fluctuations of the <4-μm size components are correlated to those of the 4–16 μm ones, and the others are correlated with each other. The particle size frequency distribution curves for dust samples in the Ebinur drainage area span a wide range of 0.3–700 μm, within the modal particle size of 10–100 μm (Fig. 3).

## Discussion

**Fig. 4:**
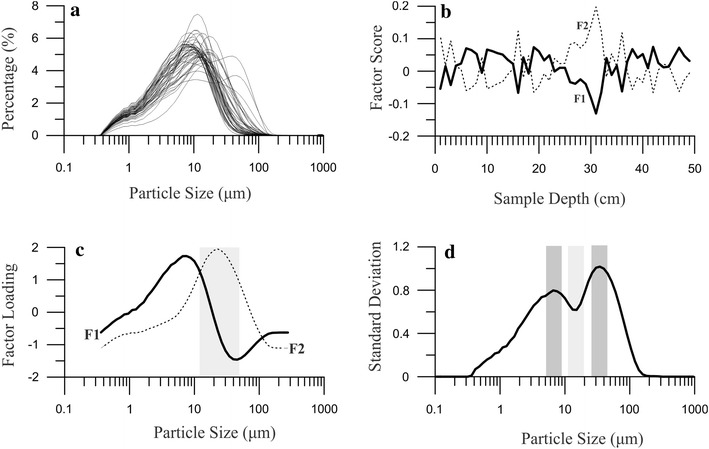
The particle size distribution of Ebinur Lake sediments (**a**), the factor score (**b**) with their factor loading coefficients (**c**) from factor analysis, and the standard deviation values versus particle size of sediment core AB01 (**d**)

Factor analysis was applied as a useful method for source apportionment and identification of the influences on sedimentary environments (Christensen and Bzdusek 2005; Sofowote et al. 2008). Factor analysis was conducted by SPSS software with variables of the particle size frequency distribution of different samples (Fig. 4a). The first (F1) and second (F2) factor accounted for 61.6 and 36.5 % of total variance, respectively. The factor-score (Fig. 4b) and factor-loading (Fig. 4c) matrices can be interpreted in terms of source profiles and contributions, respectively. The coarse particle size fraction (F2) was combined with the moderate-to-coarse grain sediments with a modal diameter of 52 μm. The modal diameter of the coarse fraction is consistent with dust samples from Ebinur drainage area, which showed that the large particle fraction of lake sediments was mainly from the drainage surface sediments.

**Fig. 5:**
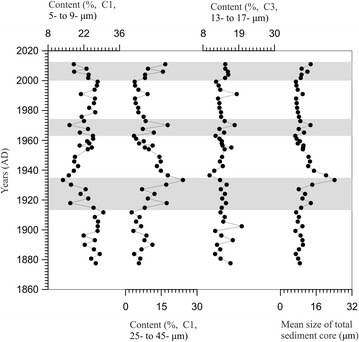
The content of sensitive grain-size components and the mean size of total sediment core

The variation of standard deviation method (Boulay et al. 2003; Sun et al. 2003) was used to identify the grain size intervals with the highest variability along a sedimentary sequence. From Fig. 4d, we can identify the intervals with the highest value of standard deviation. Figure 4d displays standard deviation values versus particle size classes of the collected sediments. Two peaks are observed at particle size intervals of 5–9 (C1) and 25–45-μm (C2), respectively. Each of two components represents a subpopulation with the highest variability along the sedimentary sequence (Fig. 5). The intermediate fraction of 13–17-μm (C3) had low standard deviation values, which indicated unconspicuous variation through time (Fig. 5). The content fluctuation of C1 is inversely correlated to those of C2, however, the fluctuation of the coarse population (F2) distribution are correlated to the mean size distribution, which shows that the coarse grain-size population mainly influences the entire variation of the sedimentary sequence. Based on the factor analysis, the coarse environmental population was from aeolian terrigenous materials. At depths of 45, 11 and 4 cm (Fig. 5) corresponding to 1915–1935, 1965–1975 and since the beginning of the 2000s, the proportions of the two populations display marked variability that be attributed to three environmental events.

**Fig. 6:**
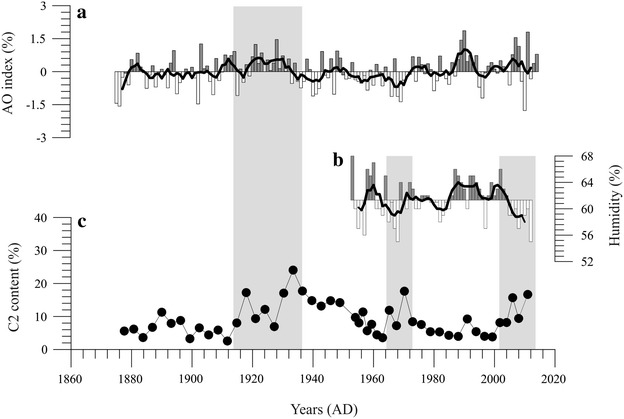
**a** The Arctic Oscillation index (Li and Wang, 2003) with five-running average. **b** The relative humidity in Jinghe with five-running average. **c** Aeolian environmental variation in Ebinur Lake inferred from the C2 contents

From ca. 1910 to the 1930s, regional climate was generally dry inferred from organic matter and its stable isotope (^13^C) in the lacustrine records of Chaiwopu Lake (Ma et al. 2013), the record of Palmer Drought Severity Index (PDSI) in the central Tianshan Mountain area (Li et al. 2006), and the tree-ring data in northern China (Liang et al. 2006). From 1950s, Modern weather station records can provide us the instrumental data. To analyze the modern humidity changes in the Ebinur drainage area, relative humidity records from the Jinghe Meteorological Station in this region (China Meteorological Data Sharing Service System, http://data.cma.cn; Fig. 6). Due to the differences in time resolution between the data of relative humidity and C2 contents, the average data were recalculated with 5-year intervals. The contents of C2 reflected the intensity of aeolian transport are negatively correlated with data of the regional humidity in Jinghe with 5-year time resolution (r = −0.613, p < 0.05). That dry climate provided abundant material basis for aeolian dust transports.

Abundant sand and dust materials are the foundation for aeolian dust transport; however, Wind provides a dynamic condition (Zhang et al. 2005). Some researchers have shown that climate change in Xinjiang has a significant teleconnection with the North Atlantic Oscillation (NAO) (Chen et al. 2006), Siberia High (Li et al. 2012a), and the Arctic Oscillation (AO) (Li et al. 2012b). In the period of the 1910s–1930s, significant variation in the in NAO, AO (Hurrell et al. 2003) and SH indices appeared (D’Arrigo et al. 2005; Jeong et al. 2011), which inferred that the climate in our study area experienced a severe change induced by the instability of the atmosphere. The wind is one of the driving forces for dust transportation (Liu et al. 2004). The AO, shown in Fig. 6 (Li and Wang 2003), reflects the tendency for the zonal winds at 35° and 55°N (Neff et al. 2008). During the high index state of the AO, surface pressure of the polar region is low, which induce the middle latitude jet stream to blow more strongly; westerly winds become stronger than normal over the northern Eurasian Continent (Sun et al. 2001). The strong westerly jet in the upper troposphere is linked to the development of cyclones and frontogenesis at low levels that can cause strong winds and provided the dynamic factor for the increase dust storm in central Asia and Xinjiang.

In order to distinguish the dynamic condition controlled the aeolian dust transport, we calculated the correlation coefficient between the AO index, and C2 contents reflected the intensity of aeolian transport. Due to the differences in time resolution between the AO index and C2 contents, the average data were also recalculated with 5-year intervals. There was not correlation between AO index and C2 contents from 1875 to 2010 (r = 0.251, N = 28); however, the AO index has a remarkable correlation to C2 contents from 1875 to 1950 (r = 0.615, N = 16, p < 0.05). Based on the above analysis, climate was dry around 1910s–1930s in this region associated with the appropriate dynamic condition, which provided the enhanced source materials and wind power for the aeolian dust transport. Since 1950s, the climate controlled the foundation of aeolian dust transport, and the aeolian dust transport won’t be increased under the humid climate.

## Conclusions

Lakes in arid areas received materials from runoff and conveyed by wind. Mathematics can be used in important ways to apply data during the in-depth study of environmental information related to sediment grain-size data when combined with instrumental records and historical documents.

With comparison of natural dust samples, the coarser particle fraction of the Ebinur Lake core sediments was mainly from aeolian transport of drainage surface sediments. The content of the 25–45-μm size class represents a component with the highest changing frequency and the coarse particle-size population mainly influences the entire variation of the sedimentary sequence.

During the last 150 years, the sediments contain significant amounts of aeolian dust and low amounts of riverine particles during the periods of 1915–1935 and 1965–1975, and since the beginning of the 2000s. The climate was dry around 1910s–1930s in this region associated with the appropriate dynamic condition, which provided the enhanced source materials and wind power for the aeolian dust transport. Since 1950s, the climate controlled the foundation of aeolian dust transport, and the aeolian dust transport won’t be increased under the humid climate.
